# Novel Design of Imputation-Enabled SNP Arrays for Breeding and Research Applications Supporting Multi-Species Hybridization

**DOI:** 10.3389/fpls.2021.756877

**Published:** 2021-12-22

**Authors:** Gabriel Keeble-Gagnère, Raj Pasam, Kerrie L. Forrest, Debbie Wong, Hannah Robinson, Jayfred Godoy, Allan Rattey, David Moody, Daniel Mullan, Tresslyn Walmsley, Hans D. Daetwyler, Josquin Tibbits, Matthew J. Hayden

**Affiliations:** ^1^Agriculture Victoria, AgriBio, Centre for AgriBioscience, Bundoora, VIC, Australia; ^2^InterGrain, Bibra Lake, WA, Australia; ^3^School of Applied Systems Biology, La Trobe University, Bundoora, VIC, Australia

**Keywords:** SNP genotyping, imputation, GWAS, genomic selection, molecular breeding, dual sample hybridization, wheat, barley

## Abstract

Array-based single nucleotide polymorphism (SNP) genotyping platforms have low genotype error and missing data rates compared to genotyping-by-sequencing technologies. However, design decisions used to create array-based SNP genotyping assays for both research and breeding applications are critical to their success. We describe a novel approach applicable to any animal or plant species for the design of cost-effective imputation-enabled SNP genotyping arrays with broad utility and demonstrate its application through the development of the Illumina Infinium Wheat Barley 40K SNP array Version 1.0. We show that the approach delivers high quality and high resolution data for wheat and barley, including when samples are jointly hybridised. The new array aims to maximally capture haplotypic diversity in globally diverse wheat and barley germplasm while minimizing ascertainment bias. Comprising mostly biallelic markers that were designed to be species-specific and single-copy, the array permits highly accurate imputation in diverse germplasm to improve the statistical power of genome-wide association studies (GWAS) and genomic selection. The SNP content captures tetraploid wheat (A- and B-genome) and *Aegilops tauschii* Coss. (D-genome) diversity and delineates synthetic and tetraploid wheat from other wheat, as well as tetraploid species and subgroups. The content includes SNP tagging key trait loci in wheat and barley, as well as direct connections to other genotyping platforms and legacy datasets. The utility of the array is enhanced through the web-based tool, *Pretzel* (https://plantinformatics.io/) which enables the content of the array to be visualized and interrogated interactively in the context of numerous genetic and genomic resources to be connected more seamlessly to research and breeding. The array is available for use by the international wheat and barley community.

## Introduction

High-density genotyping arrays that simultaneously interrogate thousands of single nucleotide polymorphisms (SNPs) have proven to be a powerful tool in genetic studies. The first generation of these have been widely used in wheat (*Triticum aestivum* L.) and barley (*Hordeum vulgare* L.) for various applications including genome-wide association studies (GWAS), characterization of genetic resources, marker-assisted breeding, and genomic selection (Joukhadar et al., 2017; Pasam et al., 2017; Balfourier et al., 2019). Continued advances in genome assembly and genotyping technologies present powerful new opportunities to continue the integration of genomics information into operational plant breeding systems and extend the potential of more academic research applications; e.g., studying genomic patterns of diversity, inferring ancestral relationships between individuals in populations and studying marker-trait associations in mapping experiments.

Chromosome-scale genome assemblies are becoming available for more and more species and this availability is expected to accelerate with international projects such as the Earth BioGenome Project^1^ which aims to sequence, catalog, and characterize the genomes of all of the eukaryotic biodiversity of the earth over the next 10 years. High quality assemblies are already available in cereal crop species, such as barley (Mascher et al., 2017; Monat et al., 2019), emmer wheat (Avni et al., 2017), durum wheat (Maccaferri et al., 2019), and bread wheat (The International Wheat Genome Sequencing Consortium [IWGSC], 2018), as well as for the diploid ancestors of wheat (Luo et al., 2017; Ling et al., 2018). These assemblies have accelerated SNP discovery and our understanding of the breeding history of wheat and patterns of genome-wide linkage disequilibrium (LD) in different germplasm pools. For example, He et al. (2019) used an exome capture array in 890 globally diverse hexaploid and tetraploid wheat accessions to discover 7.3M varietal SNPs and investigate the role of wild relative introgressions in shaping wheat improvement and environmental adaptation. Pont et al. (2019) exome-sequenced a worldwide panel of 487 accessions selected from across the geographical range of complex wheat species to explore how 10,000 years of hybridization, selection, adaptation, and plant breeding have shaped the genetic makeup of modern bread wheat. Similarly, Mascher et al. (2019) discovered almost 15M varietal SNPs from exome sequence generated for 96 two-row spring and winter barley accessions, a subset of which was used to investigate the extent and partitioning of molecular variation within and between the two groups.

While SNP discovery using whole genome sequence data is currently limited to a relatively small number of wheat and barley accessions, this situation is expected to rapidly change as sequencing costs continue to decrease. For example, 4M group 7 chromosome SNPs from 16 bread wheat accessions (Lai et al., 2015) and 36M whole genome SNPs from 18 bread wheat accessions (Montenegro et al., 2017) have previously been reported. The more recent publication of the whole genome sequence assemblies for 15 hexaploid wheat varieties from global breeding programs (Walkowiak et al., 2020) provides additional new resources for *de novo* whole genome SNP discovery and investigating structural variation within the wheat genome. In barley, Hill et al. (2020) used a combination of data sources including low coverage whole genome sequence of 632 genotypes representing major global barley breeding programs to investigate genomic selection signatures of breeding in modern varieties.

Increasing genomic resources and increased understanding of global and local population structure (Joukhadar et al., 2017) enable a shift from higher- to lower-density genotyping assays as a basis for undertaking genetic analyses for trait dissection and mapping. Where high-density data is still required, imputation can be effective to accurately infer higher marker density. Imputation uses statistical approaches to fill missing genotype data and increase low-density genotype data to genome-wide high-density data (Money et al., 2015). Imputation has been shown to increase the power of the detection of marker-trait associations in GWAS (Jordan et al., 2015; Fikere et al., 2020) and genomic selection (Nyine et al., 2019). Currently, hybridization-based SNP arrays are better suited for imputation, compared to genotyping-by-sequencing (GBS) approaches, due to their lower missing data rates and higher genotype calling accuracies (Rasheed et al., 2017; Elbasyoni et al., 2018).

To date, several hybridization-based SNP genotyping arrays providing genome-wide coverage have been developed for wheat and barley. Cavanagh et al. (2013) developed an Illumina iSelect array that genotyped 9,000 SNPs. The same technology was used a year later to design an array that assayed 90,000 SNPs (Wang et al., 2014), which was subsequently used to derive a breeder-oriented Infinium 15K array (Soleimani et al., 2020). Winfield et al. (2016) reported an Affymetrix Axiom 820K SNP array, which was also subsequently used to derive an Axiom 35K Wheat Breeders’ array that targeted applications in elite wheat germplasm (Allen et al., 2015). These genotyping arrays were largely based on genome sequence fragments from early Roche 454 and Illumina assemblies, or from exome capture sequence, and were generally enriched for gene-associated SNPs. More recently, Rimbert et al. (2018) reported an Axiom 280K SNP array based on content derived from the intergenic fraction of the wheat genome, which to date has been poorly exploited for SNP, while Sun et al. (2020) described an Axiom 660K array based on genome-specific markers from hexaploid and tetraploid wheat, emmer wheat, and *Aegilops tauschii*. In barley, two Infinium iSelect genotyping arrays comprising 9K and 50K SNPs have been reported (Comadran et al., 2012; Bayer et al., 2017).

While SNP genotyping arrays provide robust allele calling with high call rates and fast sample turnaround (typically about 3 days), they have high setup costs. The latter has presented significant challenges for the development of SNP arrays that can comprehensively serve both research and breeding applications; researchers have traditionally preferred high SNP density (which creates a high genotyping cost per sample but low cost per data point), while breeders typically only want a minimally sufficient marker density. This challenge drove us to develop a general approach to SNP array design that specifically takes into consideration the need for low-cost genotyping across a wide range of research and breeding applications, with the aim to seamlessly connect research to breeding.

Here, we present the design methodology and an example of its implementation in the Illumina Infinium Wheat Barley 40K SNP array Version 1.0, a new and highly optimized genotyping platform containing 25,363 wheat-specific and 14,261 barley-specific SNP, the vast majority of which behave as easily scored, single-copy biallelic markers. The SNP content was carefully selected to enable accurate imputation to high SNP density in globally diverse wheat and barley germplasm, as well as within the more restricted germplasm pools of breeding programs. The array is well connected to markers on other commonly used SNP arrays and to many existing genomic resources and provides high utility in research and breeding from germplasm resource characterization, GWAS, and genetic mapping to tracking introgressions from different sources, marker-assisted breeding, and genomic selection. In addition, the SNPs have been selected to enable joint hybridization of wheat and barley samples in the same assay, potentially halving costs for large-scale deployment. The array is available for use by the international wheat and barley community and is supported by the web tool, *Pretzel* (Keeble-Gagnère et al., 2019)^2^.

## Materials and Methods

### Germplasm and Genomic Resources

Single nucleotide polymorphism genotypes for 1,041 exome-sequenced bread wheat accessions were used to select content for the Infinium Wheat Barley 40K SNP array. The accessions included 790 previously reported by He et al. (2019) to capture global wheat (*T. aestivum*) diversity, an additional 149 accessions selected from the global collection contained in the associated VCF file^3^ to expand the diversity captured and 102 historical breeding lines from the InterGrain commercial wheat breeding program^4^. The first two sets of accessions maximally captured genetic diversity among 6,087 globally diverse wheat accessions comprising landraces, varieties, synthetic derivatives, and novel trait donor lines (He et al., 2019). The additional 149 accessions were selected to capture genetic diversity within synthetic derivative germplasm derived from crossing 100 primary synthetics (derived from interspecific hybridization of durum wheat with *Ae. tauschii*) to three Australian varieties: Yitpi, Annuello, and Correll (Ogbonnaya et al., 2007). The latter two sets of accessions were exome-capture sequenced as described in He et al. (2019). SNP discovery was performed using the first two sets of accessions and the resulting SNP list was used to call SNP genotypes across all accessions.

The Infinium 90K wheat SNP genotypes reported by Maccaferri et al. (2019) for a globally diverse tetraploid wheat collection of 1,856 accessions comprising wild emmer (*T. turgidum* ssp. *dicoccoides*), domesticated emmer (*T. turgidum* ssp. *dicoccocum*), and *T. turgidum* genotypes including durum landraces and cultivars were used to select tetraploid wheat specific SNP.

A georeferenced landrace collection of 267 exome-sequenced barley accessions, including 2- and 6-rowed *H. vulgare* landraces as well as *H. spontaneum* (Russell et al., 2016), and 117 whole genome sequenced accessions representing historical breeding lines from the InterGrain commercial barley breeding program were used to select the content for the SNP array.

### SNP Discovery

In wheat, SNP discovery and genotype calling were performed as described by He et al. (2019), against IWGSC RefSeq v1.0 (The International Wheat Genome Sequencing Consortium [IWGSC], 2018). After filtering for > 60% call rate and > 1% MAF, 2.04M SNPs were used for LD analysis. To filter for nucleotide variation originating from *Ae. tauschii*, D-genome-specific SNP that had a MAF > 0.1 in the synthetic derivative wheat and MAF < 0.1 in the globally diverse wheat collection were identified. In addition, the top 2% of D-genome SNPs that showed differential allele frequencies between these two groups based on F_st_ values (Weir and Cockerham, 1984) were selected. From these two SNP sets, SNP uniformly distributed across the D-genome were selected for inclusion as SNP content.

In barley, SNP discovery was performed as described by He et al. (2019) using the exome sequence data published by Russell et al. (2016), against Morex v1.0 (Mascher et al., 2017). Following the removal of *H. spontaneum-*like accessions based on principal component analysis (PCA) clustering (which left 157 *H. vulgare*-like accessions), the resulting SNP list was used to call SNP genotypes in the 120 InterGrain historical breeding lines. After filtering for > 60% call rate and > 5% MAF (a higher cut-off was used in barley due to the smaller reference population), 932,098 SNPs were used for LD analysis.

### Linkage Disequilibrium Analysis

Linkage disequilibrium analysis for the filtered SNP was performed using PLINK (Purcell et al., 2007) at the chromosome level within each species with a maximum window size of 2 Mb; i.e., all the SNPs in a tag SNP set (see “Results” for definition) had to be within a 2 Mb window. The squared correlation coefficient (*r*^2^) based on the allele frequency in the global barley or wheat diversity panel (excluding the synthetic derivatives) between two SNPs was considered as a measure of LD.

### Choice of SNP Probe Designs

To maximize the number of SNPs assayed for a given number of probes on the bead chip array, A/T and C/G variants (Infinium Type I SNP which require two probes) were avoided. To maximize SNP scorability and genotype calling accuracy, polymorphism underlying the 50-mer oligonucleotide SNP probe sequences were also avoided as they are known to cause shifts in SNP cluster position (Wang et al., 2014). For tagging SNPs (tSNPs), the probe sequences were required to align uniquely to the target genome and not aligned to the other genome; i.e., a wheat SNP probe had to align uniquely to the wheat genome and not to the barley genome, and vice versa. Finally, an Illumina Design Tool score of ≥ 0.6 was required for a probe to be included as array content. A relaxed set of criteria was also used (to tag SNP sets otherwise missed) which allowed up to three alignments to the target genome.

### Selection of Tagging SNP for Imputation

A custom algorithm was used to select tSNP tagging LD blocks in each of the global collections and to facilitate imputation from the density of the SNP array. In brief, for each chromosome, the algorithm iteratively selected the most informative tSNPs passing all filters (based on its *r*^2^ value from the LD analysis), removed all SNPs linked to the selected tSNPs from the remaining list of SNPs, as well as all SNPs linked to any SNP in the selected tSNP set to avoid directly tagging any SNP at *r*^2^ ≥ 0.9 more than once, before repeating the process until a target number of tSNP was reached. This process ensured that the set of tSNP selected was the minimum set required to tag the most SNPs at *r*^2^ ≥ 0.90. Specifically, for a given set of SNPs *S* = {*s_1_*,*s*_2_,…} and function *r*^2^(*s*_*i*_,*s*_*j*_) defining the *Pearson correlation coefficient squared* ∀*s*_*i*_,*s*_*j*_ ∈ *S*, we define the tSNP set for *s_i* at *q* to be:


$$T_{s_{i}}^{q}=\left\{s_{j}\in S|r^{2}\,\left(s_{i},s_{j}\right)\geq q\right\}.$$

Rename the *T^q*_*s_i*_ and define $T_{s\,o\,r\,t\,e\,d}^{q}={\left(T_{s_{j}}^{q}\right)}_{j=1}^{n}=T_{s_{1}}^{q},T_{s_{2}}^{q},T_{s_{3}}^{q},\ldots$ where $i\geq j=>|T_{s_{i}}^{q}|\leq |T_{s_{j}}^{q}|.$

In other words, *T^q*_*sorted*_ is an ordering of equivalent SNP sets, monotonically decreasing in size.

Let *F*⊂*S* be a subset of filtered SNPs. Define $F\,\left(T_{s\,o\,r\,t\,e\,d}^{q}\right)=\left\{T_{s_{j}}^{q}|s_{j}\in F\right\}$.

We define $T_{s\,o\,r\,t\,e\,d}^{q}-T_{s_{i}}^{q}=\left\{{\left(T_{s_{j}}^{q}\right)}_{s_{j}\in S}|T_{s_{i}}^{q}\cap T_{s_{j}}^{q}=\emptyset \right\}$, *head*(*L*) to be the first element of the ordered sequence *L*, and select $\left(T_{s_{i}}^{q}\right)$ = *s_i*.

The algorithm is then:


$$S_{i\,m\,p}\leftarrow \emptyset$$

$$T\leftarrow h\,e\,a\,d\,\left(F\,\left(T_{s\,o\,r\,t\,e\,d}^{q}\right)\right)$$

while⁢|T|≥m:

$$\,S_{i\,m\,p}\leftarrow S_{i\,m\,p}\cup \left\{s\,e\,l\,e\,c\,t\,\left(T\right)\right\}$$

$$\,T_{s\,o\,r\,t\,e\,d}^{q}\leftarrow T_{s\,o\,r\,t\,e\,d}^{q}-T$$

$$\,T\leftarrow h\,e\,a\,d\,\left(F\,\left(T_{s\,o\,r\,t\,e\,d}^{q}\right)\right)$$

For example, the above applied with *q* = 0.9,*m* = 10 defines the first iteration of tSNP selection.

To guard against possible loss of imputation accuracy due to SNP assays failing to provide reliable genotype calls, a level of redundancy was included in the tSNP sets for wheat and barley. Specifically, three tSNPs were chosen when the number of SNPs tagged was ≥ 50 and two tSNPs were selected when the number of SNPs tagged was ≥ 20. Single tSNP were selected when they tagged at least 10 SNPs. Some SNP sets could not be tagged because no probe passed all the filters; in this case, we ran the algorithm on the remaining sets allowing for SNP passing relaxed filters (up to three hits to the target genome were allowed). In addition, tSNPs were selected to tag genomic regions that had sparse SNP coverage but high LD; i.e., tagging < 10 SNP within windows larger than 500 Kb in wheat and 1Mb in barley. Finally, SNPs were selected in regions still lacking SNPs after the previous steps.

### Optimization of SNP Content

To ensure broader applicability of the SNP array in research and breeding, the content included SNP selected to specifically interlink germplasm resources, such as the 19,778 domesticated barley accessions with GBS genotypes described by Milner et al. (2019). It also included SNP probes designed to interrogate published trait-linked markers in wheat and barley. Designs for these markers were based directly on published sequences or from the alignment of published primers or flanking sequences and inference of the targeted nucleotide variation. For all trait-linked markers, the best probe design was selected based solely on the Illumina quality score. Due to the difficulty of designing SNP probes targeting known alleles of phenology genes, we selected 293 exome SNPs around the genes reported by Shi et al. (2019).

### Imputation

The wheat and barley global diversity sets were used as reference haplotypes for imputation. For wheat, accessions clustering with the synthetic derivatives in a PCA analysis were excluded. For barley, only samples with < 20% missing data were used. In both species, missing data were filled in using Beagle 4.1 (Browning and Browning, 2007) and phased with Eagle 2.4.1 (Loh et al., 2016). In total, 868 and 155 wheat and barley lines were used as reference haplotypes.

In wheat, SNP coordinates were converted to IWGSC v2.0 pseudomolecules^5^ (Zhu et al., 2021) before imputation. After transfer into the v2.0 assembly, there were 18,521 tSNPs before imputation, with 630,058, 549,003, and 352,947 tagged at *r*^2^ ≥ 0.50, 0.70, and 0.90, respectively.

To assess the accuracy of imputation into globally diverse germplasm, 100-fold cross validation was performed. A random subset of 100 wheat (or 10 barley) lines had their true genotypes masked, leaving only the tSNP. The remaining lines were then used as the reference population with Minimac3 software (Das et al., 2016) to impute back the missing genotypes for three different target SNP sets: the set of SNPs tagged at *r*^2^ ≥ 0.50, 0.70, and 0.90. The imputation accuracy for each line, measured as both correlation squared and concordance between the actual and imputed genotypes, was calculated from 100 repetitions of this process in each of wheat and barley. The correlation squared metric used was the Pearson correlation coefficient squared (*r*^2^) between SNPs called in both genotypes being compared, while concordance was measured as the fraction of SNPs in agreement with those called in both genotypes being compared.

### Genome-Wide Association Studies

Genome-wide association studies were performed using the GCTA software (Yang et al., 2011) using a mixed linear model with the SNP matrix fitted as a fixed effect and genomic relationship matrix (GRM) as a random effect. The GRM is a covariance matrix from the SNP information for each sample. Phenotype data for awned status (scored as a presence-absence trait) in 355 wheat accessions and row type (scored as two- or six-rowed) in 121 barley accessions were used. The number of SNPs used in the wheat GWAS, after transfer into the IWGSC v2.0 assembly (see imputation section above), were 18,515 (selected tSNP), 548,864 (imputed tSNP) and 1,086,408 (exome). The number of SNPs used in the barley GWAS were 13,518 (selected tSNP), 359,752 (imputed tSNP), and 1,719,837 (exome). An arbitrary threshold *P*-value of 1×10^–5^ was used as the significant threshold for declaring a marker-trait association.

### SNP Assay and Genotype Calling

Samples were assayed following the protocol for Infinium XT bead chip technology (Illumina Ltd., CA, United States). SNP clustering and allele calling was performed using GenomeStudio Polyploid software (Illumina Ltd., CA, United States) using the Illumina-supplied wheat or barley SNP manifest file. The custom genotype calling pipeline described by Maccaferri et al. (2019) was also used.

### Principal Component Analysis and Plots

Figures and plots were produced in R 3.6.1^6^ and ggplot2 (Wickham, 2016). For PCA plots, SNPRelate 1.20.1 (Zheng et al., 2012) was used.

## Results

### Overview of the Design Approach

The central idea of the design concept is to exploit LD using the *r*^2^ measure to define sets of SNPs that can be considered equivalent; for a given SNP (referred to as a tSNP), we define its tag SNP (or tSNP) set as the set of SNPs tagged by this SNP at *r*^2^ ≥ 0.9. This metric provides a measure of equivalence as well as a natural ranking of SNP by their informativeness, as defined by the size of their tSNP set. We assume that the relationship is symmetrical; i.e., if SNP A is in the tSNP set of SNP B, then SNP B should be in the tSNP set of SNP A. The original set of SNPs are then filtered using technology and application-specific criteria (see section “Materials and Methods”) while maintaining connectivity to SNPs that fail the filters via the tSNP that pass the filters.

**FIGURE 1:**
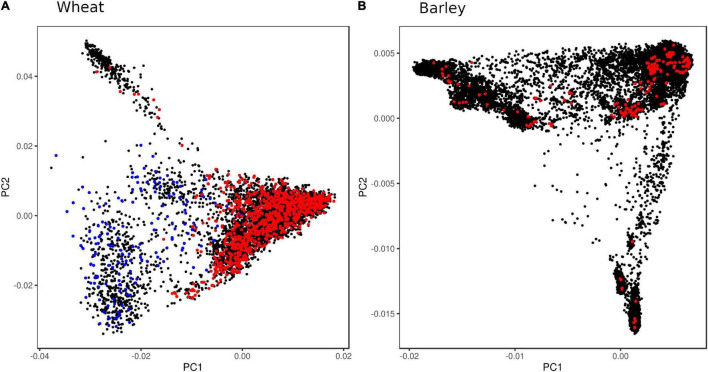
Principal component analysis (PCA) plots showing genetic diversity of wheat and barley accessions used for SNP discovery. **(A)** About 6,087 wheat accessions were genotyped with the iSelect wheat 90K SNP array (Wang et al., 2014) (black), exome-sequenced accessions used for linkage disequilibrium (LD) analysis (red), and synthetic derivative accessions capturing D-genome diversity (blue); and **(B)** 19,778 barley accessions genotyped with GBS (Milner et al., 2019) (black), with exome-sequenced accessions used for LD analysis (red).

To design a genotyping array that has broader applicability in research and breeding, the SNPs should be discovered in diverse germplasm to avoid ascertainment bias (since LD is population-dependent) and with sufficient density to produce large tSNP sets. The latter helps ensure that at least one SNP in a tSNP set will pass all the design filters in most instances. Here, we used a globally diverse set of barley landrace accessions and a globally diverse set of wheat accessions that included landraces, varieties, novel trait donors, and historical breeding lines (Figure 1). For array designs that are focused only on breeding applications, SNP discovery should aim to capture the genetic diversity within the breeding germplasm pool.

A novel selection algorithm (described in “Materials and Methods”) is then used to select SNPs that maximize LD capture, while minimizing the number of SNPs assayed on the array, using only SNPs that pass the design filters.

The design concept can be applied to any animal or plant species. In addition to this set of SNPs, utility in research and breeding can be further enhanced by including context-relevant SNPs, such as trait-linked markers and markers that link germplasm resources across different genotyping technologies. The approach used to design the Wheat Barley 40K SNP array is summarized in Supplementary Figure 5.

### SNP Discovery and Filtering

Filtering for a minimum minor allele frequency (MAF) of 1% and maximum missing rate of 40% using the 8,869,370 wheat SNP published in He et al. (2019) resulted in 2,037,434 high quality SNPs for downstream analysis. Of these, 122,799 SNPs had at least one array probe that passed all design filters. In barley, filtering of the 1,843,823 SNPs identified from our processing of exome capture sequence from the accessions from Russell et al. (2016) for MAF > 5% and missing rate < 40% resulted in 932,098 high quality SNPs for downstream analysis, of which 119,633 SNPs had at least one array probe passing all the filters. The filtered SNP matrices used in the subsequent analysis are available at https://dataverse.harvard.edu/dataverse/WheatBarley40k_v1.

### Linkage Disequilibrium Analysis and Selection of Tagging SNP for Imputation

Based on LD values of *r*^2^ ≥ 0.9, a total of 1.07M wheat and 413,508 barley high quality SNPs were singletons; i.e., they had no SNP within 1Mb up and downstream with *r*^2^ ≥ 0.9. These SNPs were either genuine singletons or categorized as singletons due to the absence of additional SNPs within the surrounding 2Mb region. As singleton SNPs can only be tagged directly, which is not feasible on a low-density array, these SNPs were not considered further for inclusion on the array.

**FIGURE 2:**
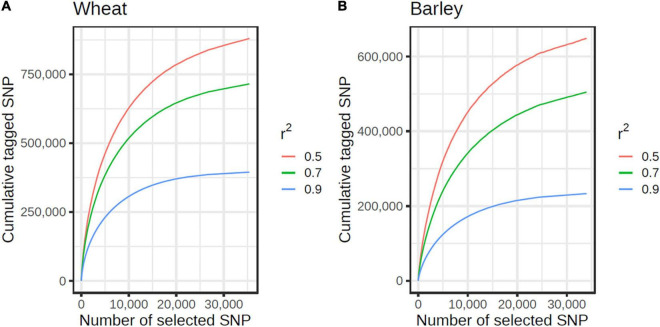
Cumulative number of SNPs tagged by tSNPs at *r*^2^ ≥ 0.9 (blue), 0.7 (green), and 0.5 (red), in wheat **(A)** and barley **(B)**. The curves are shown until the first singleton SNP (at *r*^2^ ≥ 0.90) is reached.

The custom selection algorithm grouped the 122,799 non-singleton wheat SNPs passing all design filters into 11,076 tSNP tagging SNP sets containing ≥ 10 SNP within a 2 Mb window. These tSNPs tagged 317,599, 538,326, and 652,476 SNPs at *r*^2^ ≥ 0.9, 0.7 and 0.5, respectively. Of the 119,633 non-singleton barley SNPs passing all filters, the selection algorithm identified 7,316 tSNPs which tagged a total of 150,096, 294,659, and 390,844 SNPs at *r*^2^ ≥ 0.9, 0.7, and 0.5, respectively. At the genome level, the rate of return per tSNP was surprisingly similar for wheat and barley and plateaued at about 15,000 tSNP at *r*^2^ ≥ 0.9 (Figure 2). However, the rate of return per tSNP varied at the chromosome level (Supplementary Figure 1).

**FIGURE 3:**
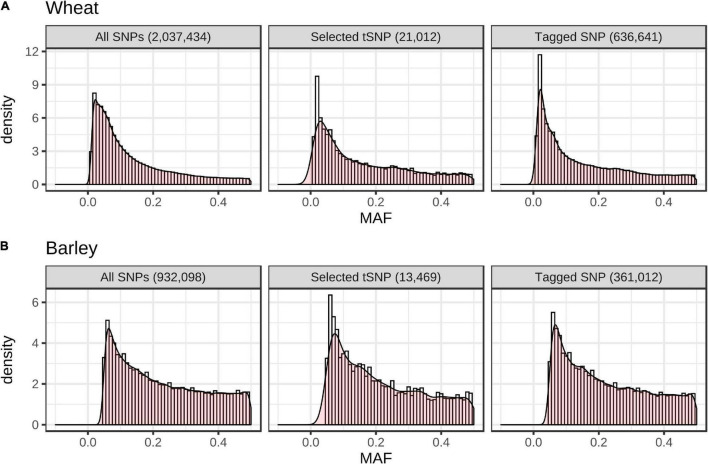
Minor allele frequency (MAF) distribution of all SNPs used for LD analysis, selected tSNPs, and the set of SNPs tagged by the tSNPs at *r*^2^ ≥ 0.70 in the globally diverse wheat **(A)** (*n* = 790) and barley **(B)** (*n* = 157) collections.

In total, 21,012 wheat and 13,469 barley tSNPs were included as content on the array. This tally includes redundant SNPs selected to guard against the possible loss of imputation accuracy due to SNP assays that might fail; SNP passing a relaxed set of filters (allowing up to three alignments to the target genome) and tagging SNP sets untaggable with the strictly filtered SNP; and SNP to tag genomic regions that had sparse SNP coverage but high LD; i.e., tagging < 10 SNPs within windows larger than 500 Kb in wheat and 1 Mb in barley. The latter SNPs are expected to support increased imputation density in these regions as higher density SNP datasets become available in the future. The wheat tSNPs tagged a total of 394,034, 636,641, and 758,452 SNPs at *r*^2^ ≥ 0.9, 0.70 and 0.50, respectively, while the barley tSNP tagged a total of 187,412, 361,012, and 471,645 SNPs, respectively. Importantly, the MAF distributions for the tSNP, tagged SNP, and filtered SNP from the globally diverse wheat and barley collections closely matched one another (Figure 3). The distribution of the selected tSNP in the wheat and barley genomes is shown in Supplementary Figure 4.

### Accuracy for Imputing Into Globally Diverse Germplasm

Cross validation (100-fold) was used to assess the accuracy for imputing from the tSNPs on the array to the sets of SNPs tagged at *r*^2^ ≥ 0.50, 0.70, and 0.90, in the globally diverse wheat and barley germplasm. This was achieved by randomly selecting 100 wheat (or 10 barley) lines and masking their true genotypes to leave only the tSNPs. Using the remaining lines as the reference population, the masked genotypes for each randomly selected line were imputed to the density of one of the target SNP sets. Accuracy was determined from the correlation squared and concordance between the imputed and actual genotypes for each wheat or barley line averaged over the occurrences of that sample within the 100 iterations.

**FIGURE 4:**
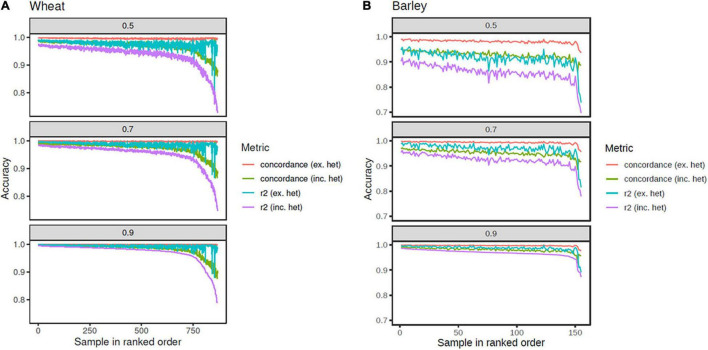
Imputation accuracy from the tSNPs on the array to the set of SNPs tagged at *r*^2^ ≥ 0.5, 0.7, and 0.9, in wheat **(A)** and barley **(B)**. Metrics plotted are correlation *r*^2^ including heterozygous calls (purple line), *r*^2^ excluding heterozygous calls (cyan line), concordance including heterozygous calls (green line), and concordance excluding heterozygous calls (orange line). The accessions are rank ordered based on the *r*^2^ including heterozygous calls.

As expected, all metrics were the highest when imputing to the set of SNPs tagged at *r*^2^ ≥ 0.90 and the lowest for those tagged at *r*^2^ ≥ 0.50 (Table 1). In wheat, only a small decrease in accuracy was observed for most accessions as the size of the tagged SNP set increased (i.e., *r*^2^ decreased), with reduced accuracy most evident in the bottom 50 accessions (Figure 4). For these accessions, the difference in accuracy (both correlation squared and concordance) between comparisons including and excluding heterozygous genotype calls was almost 10%, suggesting the possibility of high error rates in the heterozygous exome SNP calls for these accessions. About 768 (88.5%) of the wheat accessions had accuracies ≥ 90% with the strictest correlation squared metric (which included heterozygous calls) for the set of SNPs tagged at *r*^2^ ≥ 0.50. When comparing only homozygous calls, the number of lines above this threshold rose to 866 (99.8%) (Figure 4).

**TABLE 1:** Average accuracies for imputing from the tSNPs on the array to the sets of SNPs tagged at *r*^2^ ≥ 0.50, 0.70, and 0.90, in wheat and barley.

	Set of SNP tagged at *r*^2^	Wheat	Barley
Correlation squared (including heterozygous calls)	0.50	93.7 (4.0)	86.0 (3.1)
	0.70	95.3 (3.8)	92.4 (2.6)
	0.90	97.0 (3.4)	96.8 (1.6)
Correlation squared (excluding heterozygous calls)	0.50	97.6 (1.3)	91.5 (2.9)
	0.70	98.7 (1.0)	96.9 (2.3)
	0.90	99.3 (0.7)	98.7 (1.3)
Concordance (including heterozygous calls)	0.50	96.9 (2.2)	92.8 (1.4)
	0.70	97.4 (2.1)	95.2 (1.2)
	0.90	98.3 (2.0)	98.1 (0.8)
Concordance (excluding heterozygous calls)	0.50	99.6 (0.2)	98.1 (0.7)
	0.70	99.8 (0.2)	99.3 (0.5)
	0.90	99.9 (0.1)	99.7 (0.2)

*Correlation squared is defined as the Pearson correlation coefficient squared (r^2^) between SNPs called in both genotypes being compared. Concordance is the fraction of SNPs in agreement between those called in both genotypes being compared. SDs are shown in brackets.*

Reduced accuracy when imputing to higher tagged SNP numbers was more pronounced in barley. A difference of 10.8% (from 96.8 to 86%) was observed between the average correlation squared (which included heterozygous calls) for the set of SNPs tagged at *r*^2^ ≥ 0.90, compared to those tagged at *r*^2^ ≥ 0.50 (Table 1). As observed in wheat, the inclusion of heterozygous calls reduced the accuracy, particularly when imputing to the set of SNPs tagged at *r*^2^ ≥ 0.50, again suggesting possible erroneous heterozygous calls in the sequence genotypes (Figure 4). The reduced accuracies observed in barley compared to wheat are also likely to be partly due to the reduced size of reference haplotypes (155 vs. 868). Accuracies in barley are likely to improve if the reference haplotype set is expanded.

**FIGURE 5:**
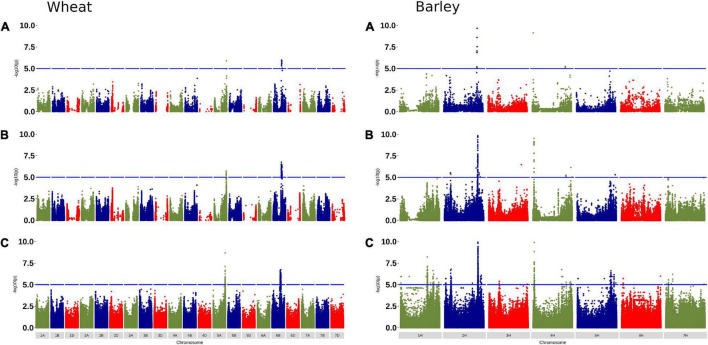
Genome-wide association study (GWAS) for awned status and row type in wheat and barley, respectively, using: **(A)** selected tSNP; **(B)** selected tSNP after imputation to the *r*^2^ ≥ 0.7 target set (the sample being imputed was removed from the reference set); and **(C)** exome SNP. Note the -log10(p) axes are scaled to 10 which resulted in the most significant SNP (38.44) for the 5A locus in wheat being out of the range on the axis for the wheat exome SNP plot.

To confirm that the selected tSNPs were useful for detecting marker-trait associations, we performed GWAS using phenotype data for awned status (scored as a presence-absence trait) in 355 wheat accessions and defining row type (scored as two- or six-rowed) in 121 barley accessions and the selected tSNPs before and after imputation. The results were compared with GWAS performed using the same phenotypic data and exome SNP genotypes (Figure 5). Significant and completely overlapping GWAS signals were observed for the three analyses performed in both wheat and barley using the different datasets. The significant SNPs in each analysis were associated with genomic regions previously reported to be associated with the traits (Russell et al., 2016; Huang et al., 2020). While the significance of the associated SNPs differed across the three analyses for each trait, the GWAS results show that the selected tSNPs effectively tag common halotype block diversity in globally diverse germplasm.

### Wheat Barley 40K SNP Array Content

The final array design comprised 34,481 tSNPs and two additional categories of context-specific SNPs (content summarized in Table 2; full details are in Supplementary Table 1).

**TABLE 2:** SNP content of the Infinium Wheat Barley 40K SNP bead chip array.

	Wheat	Barley	Total
Tagging SNP for imputation	21,012	13,469	34,481
Trait associated SNP	427	178	605
SNP linking germplasm resources	3,924	614	4,538
**Total number of SNP**	**25,363**	**14,261**	**39,624**

The first context-specific category included 2,609 SNPs from the Infinium wheat 90K SNP array (Wang et al., 2014) that were selected based on allele differentiation to tag tetraploid wheat (A- and B-genome) diversity and to clearly delineate tetraploid wheat from other types of wheat, as well as to distinguish tetraploid species and subgroups from one another. The SNPs comprised the following four classes: (1) differentiating SNPs that represent the top 2% F_st_ values in the study by Maccaferri et al. (2019) between the four subgroups of tetraploid species: wild emmer (*Triticum turgidum* ssp. *dicoccoides*), domesticated emmer (*T. turgidum* ssp. *dicoccocum*), and durum (*T. turgidum*) landraces, and durum cultivars; (2) subgroup-specific private SNPs that showed a MAF ≥ 0.1 in one of the subgroups and were either monomorphic or showed a MAF < 0.05 in the other subgroups; (3) subgroup-specific high MAF SNPs that were present at ≥ 0.3 MAF in any one of the subgroups; and (4) neutral SNPs that did not show any signatures of selection, were polymorphic in all subgroups and showed an overall MAF of ≥ 0.4. The ability of these SNPs to reliably differentiate the tetraploid species subgroups as efficiently as the Infinium wheat 90K array is shown in Supplementary Figure 2. The distribution of these SNPs across the A- and B-genomes of wheat is shown in Supplementary Figure 4.

The second category included 1,206 exome SNPs tagging *Ae. tauschii* (D-genome) diversity present in backcross synthetic derivatives that originated from crosses involving 100 primary synthetic parents, which were selected for phenotypic and genetic diversity among approximately 400 primary synthetics developed at CIMMYT and imported to Australia in 2001. Each of the 100 primary synthetic parents was derived from a different *Ae. tauschii* accession. SNPs tagging diversity in *Ae. tauschii* were selected to provide high genome coverage in the D-genome (Supplementary Figure 4). They were also selected to clearly delineate bread wheat from other types of wheat. The SNP comprised two classes: (1) differentiating SNPs that represent the top 2% F_st_ values between the global diversity wheat and synthetic derivative collections; and (2) D-genome diversity from *Ae. tauschii* that showed a MAF ≥ 0.1 in the synthetic derivative collection and MAF ≤ 0.1 in the globally diverse wheat collection. The ability of these SNPs to reliably differentiate synthetic wheat from common wheat as efficiently as the Infinium wheat 90K array is shown in Supplementary Figure 3.

The final category included linked SNPs for key breeding traits and SNPs linking major germplasm resources genotyped with different technologies. In total, 457 wheat and 178 barley SNPs corresponded to published trait-linked markers as well as 109 SNPs associated with agronomically important genes reported in published GWAS studies (Sun et al., 2017; Wang et al., 2017) (Supplementary Table 1). Another 614 SNPs provide a direct link to 19,778 GBS genotyped domesticated barley accessions (Milner et al., 2019).

### Assay Performance–Single Sample Hybridizations

A limitation of hybridization-based genotyping arrays is that their oligonucleotide probes hybridize both to the targeted locus and its homologs and paralogs, if present (Cavanagh et al., 2013; Wang et al., 2014). Consequently, the ratio of allele-specific fluorescent signals observed for an assay depends on the copy number of the locus in the genome, with increasing copy number reducing the allele-specific fluorescent signal ratio and separation of SNP allele clusters. Further, SNP assay scorability and genotype calling can be confounded by the presence of mutations that modify oligonucleotide annealing such that different cluster patterns are observed across germplasm (Wang et al., 2014). An ideal assay design for a hybridization-based genotyping array is therefore an oligonucleotide probe that binds at only one locus in the genome and has no known nucleotide variation underlying the probe hybridization site. Theoretically, this should ensure three distinct clusters corresponding to the genotypic states (REF, HET, and ALT) expected from a single-copy biallelic SNP. The increasing availability of genomic resources now allows for this historical problem to be addressed. Hence, we used the combination of reference genome assemblies and genotypic data for large globally diverse wheat and barley collections to specifically target the design of single copy biallelic SNP assays.

**FIGURE 6:**
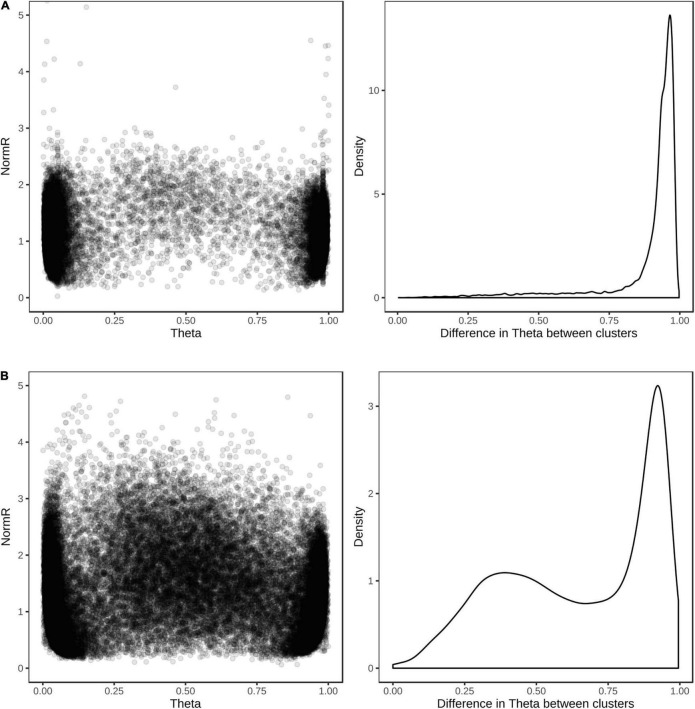
Cluster positions and theta separation of SNP in single sample hybridization assays. Scatter plot of cluster positions (left) and density plot of the difference in theta value between REF and ALT clusters (right) for **(A)** 14,261 barley and **(B)** 24,598 wheat SNP revealing polymorphism in the globally diverse wheat and barley populations.

For the purpose of evaluating the performance of the array, the wheat and barley diversity populations were used to define cluster positions for SNP genotype calling. The vast majority (98%) of the 39,654 SNP assays on the array produced scorable cluster patterns when hybridized with a barley or wheat sample; about 91% (12,949/14,261) of the barley and 83% (20,090/24,598) of the wheat SNP assays could be reliably scored as single-copy biallelic markers, with the REF and ALT clusters having theta values close to 0 and 1 in GenomeStudio SNP plots (Figure 6). While the remaining SNP could typically be reliably scored as biallelic markers, they showed cluster compression indicative of multiple loci. Few assays showed complex clustering patterns, indicating the success of designing probes without any underlying polymorphism. About 5 and 7% of wheat and barley assays showed a clustering pattern typical for the presence of a null allele. The occurrence of assays not behaving as single-copy biallelic markers reflects current knowledge gaps for structural variation in the genomes of wheat and barley including both copy number variation and presence-absence variation (Wang et al., 2014; Balfourier et al., 2019; Walkowiak et al., 2020).

The concordance between called and actual genotypes was exceptionally high for both wheat and barley. The average genotype concordance and correlation squared were 99.5 and 98.1%, respectively, in wheat when heterozygous genotype calls were excluded, and 97.6 and 95.7%, respectively, when heterozygous calls were included. Similarly, 99.8% concordance and 99.2% correlation squared were observed in barley when heterozygous calls were excluded, and 98.2 and 97.2% were observed with heterozygous calls included. The average missing data rates were 4.8 and 3.8% in wheat and barley, respectively.

### Assay Performance—Dual Sample Hybridizations

The design process specifically aimed to select species-specific SNP probes and thus it should be theoretically possible to jointly hybridize a wheat and barley sample to the same bead chip array (dual hybridization) without the loss of genotype calling accuracy. Cross-hybridization between species is expected to confound genotype calling accuracy by creating shifts in SNP cluster positions and/or complex clustering patterns that cannot be easily scored.

To evaluate the assay performance of a dual hybridization, samples from the InterGrain commercial barley and wheat breeding programs were used to define cluster positions and call SNP genotypes for 576 dual hybridization assays. The same samples were also assayed in single sample hybridization assays to enable genotype calling accuracy between dual and single hybridization assays to be directly compared.

Most of the barley and wheat SNPs in dual hybridization assays produced scorable cluster patterns. Shifts in cluster positions were observed, which indicated either that some oligonucleotide probes showed a degree of cross-species hybridization or that deviation from the standard amount of sample DNA (200 ng per sample) recommended for the bead chip assay affected signal-to-noise. Through empirical testing, we found that the quantity of genomic DNA per sample was a major factor causing shifts in cluster position (data not shown) and could be minimized by adjusting the input DNA for each sample to match the ratio of the genome size for each species; e.g., 200 ng barley DNA and 600 ng wheat DNA; the bread wheat genome is about three times larger than that of barley.

**FIGURE 7:**
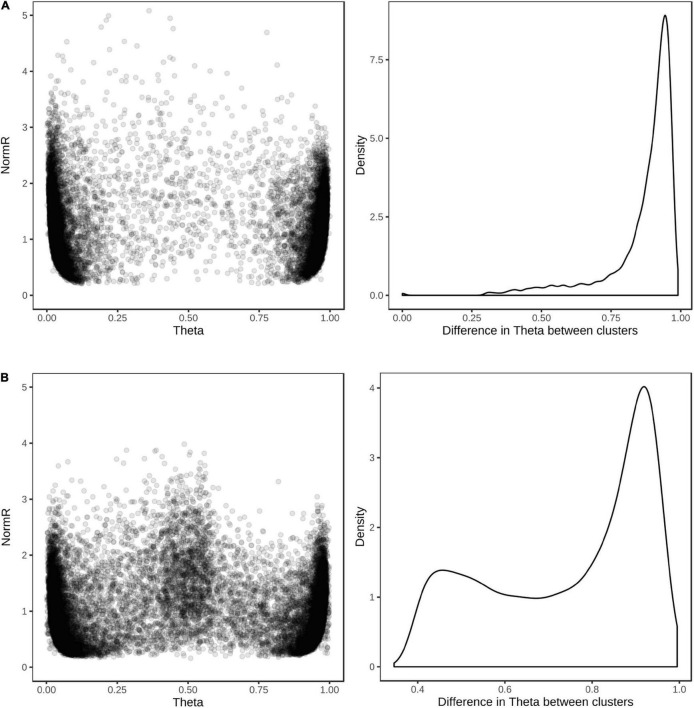
Cluster positions and theta separation of SNPs in dual hybridization assays. Scatter plot of cluster positions (left) and density plot of the difference in theta value between REF and ALT clusters (right) for **(A)** 9,826 barley and **(B)** 9,118 wheat SNP revealing polymorphism among 576 wheat and barley breeding lines.

For the purpose of assessing genotype calling accuracy for dual hybridization assays, only SNPs that revealed polymorphism among the 576 wheat and barley samples assayed were considered. Of the 9,826 barley and 9,118 wheat SNPs showing polymorphism, the vast majority were easily scored as biallelic markers and had good cluster separation, indicating that oligonucleotide probe cross-species hybridization was minimal (Figure 7). The average concordance between genotypes calls for the same wheat and barley samples in single and dual sample hybridization assays were 99.9, 96.7, and 99.8%, for the REF, HET, and ALT alleles, respectively. The average missing data rate across the wheat and barley samples was similar for both assay types, with 4.7 and 2.0% in dual and single hybridization assays, respectively.

## Discussion

High-throughput, low-cost and flexible genotyping platforms are required for both research and breeding applications. Compared to GBS and PCR-based marker systems, array-based genotyping platforms are highly commercialized and highly customizable, both for the number of markers and the samples assayed. They also have low genotype error and missing data rates compared to GBS technologies (Rasheed et al., 2017). Consequently, SNP arrays are widely utilized and several low-density SNP genotyping arrays have been developed for wheat and barley. Here, we described a novel approach that is applicable to any animal or plant species for the design of cost-effective, imputation-based SNP genotyping arrays with broad utility and that support the hybridization of multiple samples to the same SNP array. The utility of the approach was demonstrated through the development of the Infinium Wheat Barley 40K SNP array.

The key difference between the Infinium Wheat Barley 40K SNP array and previously reported array-based genotyping assays is a paradigm shift in the logic underpinning its design. To date, commonly used low-density genotyping arrays are comprised of the most scorable and informative markers from higher density arrays. For example, the Infinium Wheat 15K SNP array (Soleimani et al., 2020) and Axiom Wheat Breeders’ 35K SNP array (Allen et al., 2015) are derived from the Infinium Wheat 90K SNP array (Wang et al., 2014) and Axiom Wheat 820K SNP array (Winfield et al., 2016). SNPs on the Infinium 90K SNP array were derived from the transcriptome sequence of 19 bread wheat accessions and 18 tetraploid accessions, while those on the Axiom 820K arrays were based on exome capture sequence from 43 bread wheat and wild species accessions representing the primary, secondary, and tertiary gene pools. While these derived low-density arrays are affordable for routine deployment in breeding and research, their content is breeder-oriented and has limited utility outside the primary gene pool of hexaploid wheat.

The design implemented in the Infinium Wheat Barley 40K SNP array is based on the hugely expanded genotypic and genomic resources now available for wheat and barley. By using these resources, we were able to identify species-specific single-copy tSNPs that capture a large proportion of the haplotypic diversity in globally diverse germplasm, and are highly scorable for accurate genotype calling, minimize ascertainment bias, and enable accurate imputation to high SNP density. In the case of wheat, this included the use of 2.04M SNPs identified from exome sequence data of 1,041 accessions selected to maximally capture genetic diversity among a global collection of 6,087 accessions genotyped using the Infinium 90K SNP array (He et al., 2019; Figure 1A). The global collection included landraces, released varieties, synthetic derivatives, and novel trait donor and historical breeding lines. For barley, this included 932,098 SNPs identified from exome sequence data of 267 accessions selected to maximally capture geographic diversity among landraces (Russell et al., 2016; Figure 1B), as well as SNPs identified from target capture sequencing of 174 flowering time-related genes performed in 895 worldwide accessions (Hill et al., 2019). The latter dataset included globally diverse cultivated and landrace germplasm.

By selecting tSNPs that enable accurate imputation of common haplotype block diversity in globally diverse germplasm, the Infinium Wheat Barley 40K array is expected to maintain power for GWAS, genetic mapping, and genomic selection (Jordan et al., 2015; He et al., 2019; Negro et al., 2019; Nyine et al., 2019). Haplotype blocks are essentially fixed stretches of DNA sequence that show little historical evidence of recombination and are effectively inherited as genetic units that are shuffled and assembled during breeding. The univariate LD metric *r*^2^ has been used in many tSNP algorithms as it is a major determinant of imputation accuracy and has a simple inverse relationship with the sample size required to detect associations in GWAS (Carlson et al., 2004; Ding and Kullo, 2007). By selecting tSNPs with an *r*^2^ ≥ 0.9 cut-off, we aimed to retain most of the information content in the original SNP set and to balance the power loss with the effort needed to compensate with increased sample numbers in downstream GWAS (∼11%; i.e., 1/0.9). This aspect of the array design was confirmed by performing GWAS for awned status in wheat and row type in barley (Figure 5). A significant advantage when using *r*^2^ is that it allows for a high degree of flexibility in the composition of the final tSNP set, thereby enabling other design criteria to be applied without compromising the overall tagging efficiency. This was especially important for implementing array design principles such as selecting species-specific, single-copy SNP targets that had no nucleotide variation underlying the probes to both maximize SNP scorability and support dual sample hybridization assays. The success of our approach was confirmed by > 97% accuracy (as measured by both correlation squared and concordance between the imputed and actual SNP genotypes) when imputing the set of SNPs tagged at *r*^2^ ≥ 0.9 (inclusive of heterozygous calls) in both wheat and barley. Importantly, imputation accuracy was also high for the set of SNPs tagged at *r*^2^ ≥ 0.5 (Table 1). To futureproof the array design, we added tSNP tagging genomic regions in wheat and barley that had sparse exome SNP coverage but high LD. We expect this content will similarly support accurate imputation to whole genome sequence once genomic resources needed to achieve this are available.

In emphasizing the design focus on selecting tSNPs for imputation, we also point out the limitations it has for fully capturing the haplotype diversity in global wheat and barley germplasm. First, we did not tag LD blocks comprised of fewer than 10 SNPs since this would have required an order of magnitude more SNP assays on the array; about 30,000 tSNP per species was required to tag about half of the non-singleton exome SNP at *r*^2^ ≥ 0.9 in each of wheat and barley (Figure 2). This presents a limitation for trait mapping using GWAS (but not genetic mapping) since trait loci located in untagged LD blocks will become increasingly harder to detect as their LD with SNPs on the array decreases. This limitation can be partly overcome by increasing the sample size but is an unavoidable consequence of low-density arrays, despite our tSNP selection algorithm ensuring that we maximized the number of SNP tagged in LD. And second, the set of SNPs and LD relationships between them is still limited by the data currently available. As exome capture sequencing assays target only 2–3% of the genome, the SNPs discovered represent just a fraction of the true SNP density. It is therefore possible that SNPs were not selected simply because the haplotype they represent was only sampled by a small number of SNP in that region and was below our selection thresholds. This limitation will only be overcome by large-scale whole genome sequencing efforts which are just beginning to become affordable for large genome-sized species. It should be noted that the LD patterns detected in this study will remain valid even with higher density sequencing and that the majority of the tagged LD haplotypes span across the captured regions and so the number of SNPs in high LD with the selected tSNPs will only increase as higher density SNP data becomes available.

An argued advantage for GBS assays is that they are free from ascertainment bias. Ascertainment bias can result in rare alleles being missed and genetic diversity being underestimated in non-ascertained populations (Clark et al., 2005), with its impact dependent on the study being undertaken. Increasing marker density and low MAF markers in GWAS boosts the power for quantitative trait loci (QTL) detection (Negro et al., 2019; Fikere et al., 2020). Chu et al. (2020) reported that very low frequency markers (MAF < 0.05) contributed to an improvement of genomic prediction accuracy in 378 winter bread wheat genotypes, and combined with the expectation that valuable novel diversity is most likely rare (Mascher et al., 2019), suggests that rare markers deserve careful consideration. Our tSNP selection algorithm prioritizes haplotypes that diverge significantly from the reference genome used for SNP discovery in order to maximize the number of SNP tagged in LD; it is agnostic to the MAF of individual SNP (beyond the MAF cut-offs of 1 and 5% in wheat and barley, respectively). Consequently, the MAF spectrum of the wheat and barley tSNPs closely resembled that observed for both the sets of tagged SNPs and the filtered SNPs in the globally diverse collections (Figure 3). Hence, we suggest that the Infinium Wheat Barley 40K array has minimal ascertainment bias. Since tagging all minor variants is not feasible using low-density arrays, a better solution is to add minor variants into future versions of the array as trait associations are discovered, essentially as we have currently done for published trait linked markers.

To drive efficiencies for large-scale genotyping in commercial breeding programs, we explored the limits of the Infinium bead chip technology. One advantage of this technology is that each oligonucleotide assay probe has a unique physical position on the bead chip. This allows for SNP arrays to be designed to genotype multiple crop species, with a user-defined number of SNPs assigned to each species. The Infinium Wheat Barley 40K array assays 25,363 SNPs in wheat and 14,261 SNPs in barley. To the best of our knowledge, multispecies SNP arrays have only been used to assay a single sample at a time. Here, we demonstrated that through careful selection of species-specific oligonucleotide probes, it is possible to jointly hybridize a wheat and barley sample to the same bead chip array, without substantial loss of genotype calling accuracy (Figure 7). The selection of such probes is facilitated by our design concept which exploits LD to identify SNPs that can be considered equivalent for the purpose of genotyping. From a deployment perspective in a commercial breeding program, dual hybridization doubles genotyping throughput, since twice as many samples can be processed given the same amount of time and resources. Dual hybridization genotyping is potentially a game changing option for the adoption of genomics technologies by breeding companies with large numbers of samples that can be coordinated into genotyping.

To ensure broad utility in research and breeding, we added SNP-content capturing genetic diversity in the secondary and tertiary gene pools of wheat. This included 2,609 SNPs from the Infinium 90K SNP array (Wang et al., 2014) tagging tetraploid wheat (A- and B-genome) diversity and clearly delineating tetraploid wheat from other types of wheat, as well as tetraploid species and subgroups from one another. Each SNP is a single copy in tetraploid wheat and has been genetically and physically mapped (Maccaferri et al., 2019). It also included 1,206 single-copy SNP tagging *Ae. tauschii* (D-genome) diversity represented in 100 primary synthetic wheats, where each primary synthetic was derived from a different *Ae. tauschii* accession. Collectively, these SNPs provide broad utility ranging from the differentiation and genetic characterization of tetraploid and synthetic wheat (as well as other secondary and tertiary gene pools of wheat) to the tracking of introgressed genomic segments during breeding. Also included are SNPs that directly link to the Infinium 90K (Wang et al., 2014) and 15K (Soleimani et al., 2020) wheat arrays to ensure connectivity with legacy genotypic datasets and research. For barley, we included 685 SNPs that overlap with SNP reported for 19,778 GBS genotyped accessions from the IPK Genebank (Milner et al., 2019) to provide a direct anchor to that resource, and 1,239 SNPs that overlap with the Infinium 50K barley SNP array (Bayer et al., 2017) which link to 21,606 common SNPs following imputation. Finally, we included trait-linked SNPs and SNP tagging GWAS signals for key breeding and research targets reported in the published literature.

The overall array design makes it ideal for a wide range of research and breeding applications, from germplasm resource characterization, GWAS and genetic mapping to tracking introgressions from different sources, marker-assisted breeding and genomic selection. Its utility is further enhanced through the web-based tool, *Pretzel* (Keeble-Gagnère et al., 2019; see text footnote 2) which enables the content of the array to be visualized and interrogated in real-time in the context of numerous genetic and genomic resources. For example, the SNPs can be visualized relative to the genetic and physical positions of other DNA marker types (e.g., SSRs, DArT), SNP on other genotyping arrays, trait loci, annotated genes, and syntenic positions in the genomes of other crops and model species. The ability to upload and visualize data in *Pretzel* allows breeders and researchers to seamlessly link and interrogate their own data in the context of publicly available datasets hosted in *Pretzel*. When combined with *Pretzel*, the Infinium Wheat Barley 40K array enables legacy and current research to seamlessly connect to breeding.

## Conclusion

In conclusion, we have described a novel approach applicable to any animal or plant species for designing cost-effective imputation-enabled SNP genotyping arrays that have broad applicability in research and industry applications (e.g., GWAS, genomic prediction, and operational breeding) and support the hybridization of multiple samples to the same array. The utility of this design approach was demonstrated through its implementation to develop a new Infinium Wheat Barley 40K SNP array. In addition to supporting broad utility in research and breeding, this array can be used as a resource to connect genetic and genomic datasets generated across germplasm pools and time. The array is further supported by the publicly available web-tool *Pretzel* and is available for purchase by the international wheat and barley community from Illumina Ltd. (CA, United States), the manufacturer of the Infinium bead chip technology.

## Data Availability Statement

Exome data used from Russell et al. (2016) and He et al. (2019) are accessible under EBI ENA project accession numbers PRJEB8044 and PRJEB31218, respectively. The filtered set of exome genotype calls for accessions and SNP underpinning the LD analysis and tag SNP selection for wheat (10.7910/DVN/5LVYI1) and barley (10.7910/DVN/CUPAXD) as well as the D-genome synthetic derivative-enriched SNP matrix (10.7910/DVN/0QEASF) are available through Dataverse at https://dataverse.harvard.edu/dataverse/WheatBarley40k_v1. Information about the status of each SNP, including tag SNP set ID and whether the SNP passed design filters, is included in the INFO column. Illumina 90k iSelect genotypes for the accessions used to select tetraploid-specific content is available at https://figshare.com/articles/dataset/Durum_Wheat_cv_Svevo_annotation/6984035 (Maccaferri et al., 2019).

## Author Contributions

RP performed LD analysis. GK-G selected tagging SNP, performed imputation analyses, and produced the final designs. KF and DW performed exome and whole genome sequencing, Infinium Wheat Barley 40K assays, and genotype calling. JT performed sequence alignments and genotype calling. HR, JG, AR, DMo, and DMu selected non-tagging SNPs and provided wheat and barley germplasm. TW, HD, JT, and MH conceived the project. GK-G and MH wrote the manuscript. All authors contributed to the article and approved the submitted version.

## Conflict of Interest

HR, JG, AR, DMo, DMu, and TW were employed by InterGrain. The remaining authors declare that the research was conducted in the absence of any commercial or financial relationships that could be construed as a potential conflict of interest.

## Publisher’s Note

All claims expressed in this article are solely those of the authors and do not necessarily represent those of their affiliated organizations, or those of the publisher, the editors and the reviewers. Any product that may be evaluated in this article, or claim that may be made by its manufacturer, is not guaranteed or endorsed by the publisher.

## Footnotes

1
https://www.earthbiogenome.org/

2
https://plantinformatics.io/

3
http://wheatgenomics.plantpath.ksu.edu/1000EC/

4
www.intergrain.com

5
https://urgi.versailles.inra.fr/download/iwgsc/IWGSC_RefSeq_Assemblies/v2.0/

6
https://www.r-project.org/

## Supplementary Material

Supplementary Figure 1 Cumulative number of SNPs tagged by tSNPs at *r*^2^ ≥ 0.90 in each chromosome in wheat and barley. Curves are shown until the first singleton SNP is reached on each chromosome.

Supplementary Figure 2 Principal component analysis (PCA) based on **(A)** 17,600 SNPs described by Maccaferri et al. (2019) from the Infinium wheat 90K SNP array and **(B)** 2,609 SNPs selected for inclusion on the Infinium Wheat Barley 40K SNP array showing differentiation among 1,856 tetraploid wheat accessions representing wild emmer wheat from North Eastern Fertile Crescent (WEW-NE), wild emmer wheat from Southern Levant Fertile Crescent (WEW-SL), domesticated emmer wheat (DEW), domesticated emmer wheat from Ethiopia (DEW-ETH), durum wheat landraces (DWL), and durum wheat cultivars (DWC).

Supplementary Figure 3 PCA based on **(A)** 37,105 called SNPs from the Infinium wheat 90K SNP array, and **(B)** 20,665 SNPs on the Infinium Wheat Barley 40K SNP array showing differentiation among bread wheat (green), synthetic derivatives (blue), and hexaploid wheat derived from crosses between bread and durum accessions (red) (number of accessions = 1,219).

Supplementary Figure 4 Distribution of selected SNP content across the wheat and barley genomes. Selected tSNPs (green), tetraploid wheat-specific SNPs shown with positions as reported in the durum genome by Maccaferri et al., 2019 (blue) and synthetic wheat-derived SNPs (red).

Supplementary Figure 5 Overview of the design approach for the Wheat Barley 40K SNP array.

Supplementary Table 1 Detailed description of Infinium Wheat Barley 40K SNP array content.
