# Supplementary figures and images for: Novel Design of Imputation-Enabled SNP Arrays for Breeding and Research Applications Supporting Multi-Species Hybridization
Source: Front Plant Sci. 2021 Dec 22;12:756877. doi: 10.3389/fpls.2021.756877 (PMC8728019; doi:10.3389/fpls.2021.756877)

## Wheat

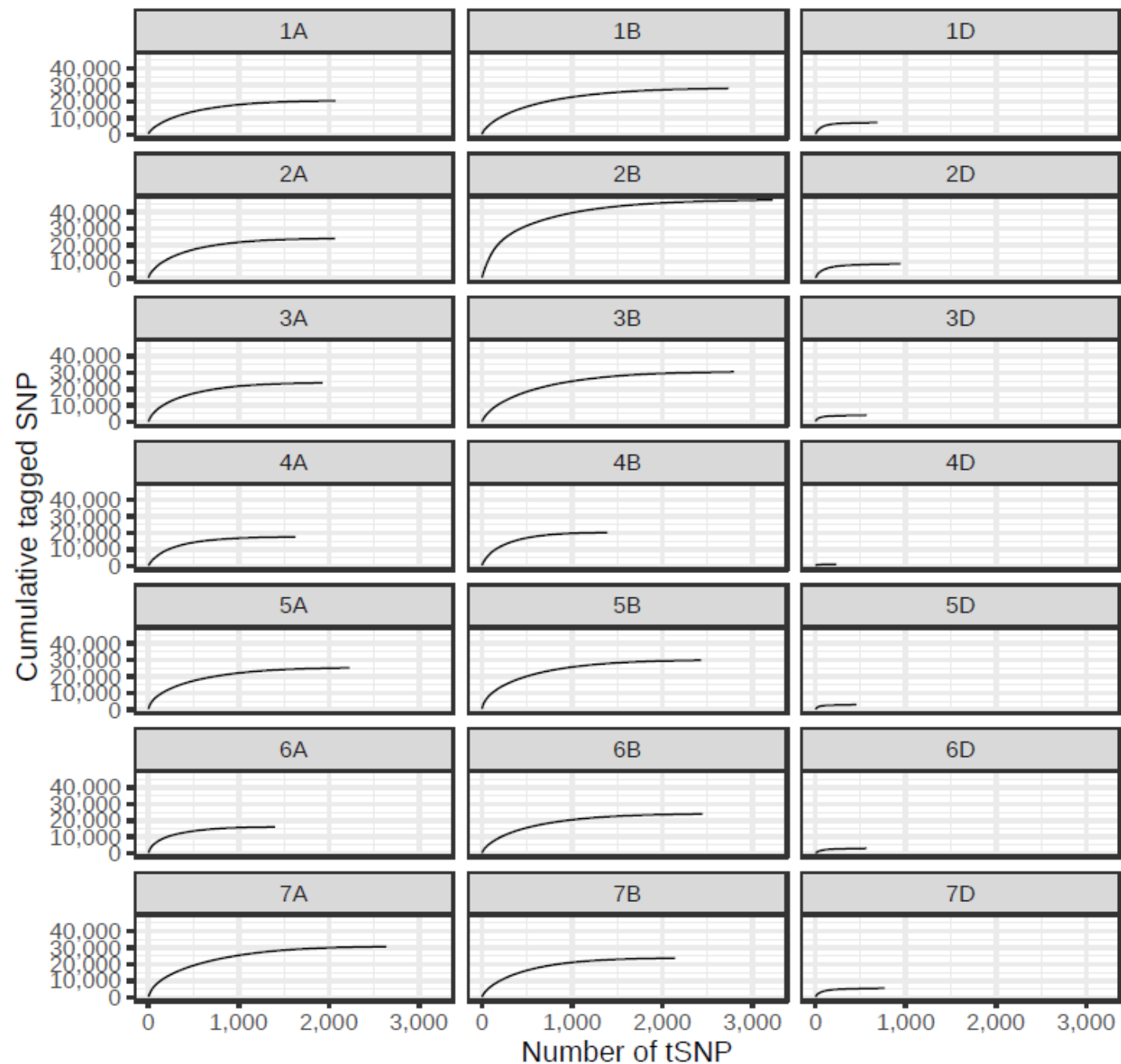

## Barley

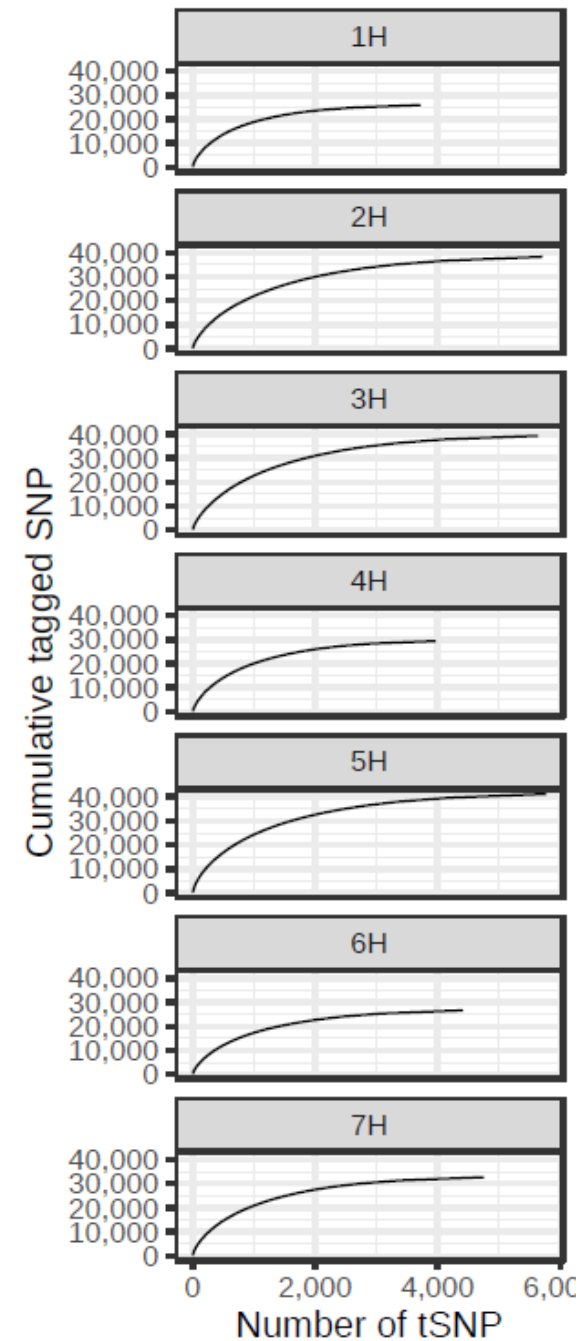

Supplement: Supplementary Figure 1 — Cumulative number of SNPs tagged by tSNPs at r2 ≥ 0.90 in each chromosome in wheat and barley. Curves are shown until the first singleton SNP is reached on each chromosome. [file Data_Sheet_1.zip › Supplementary Figure 1.PDF]

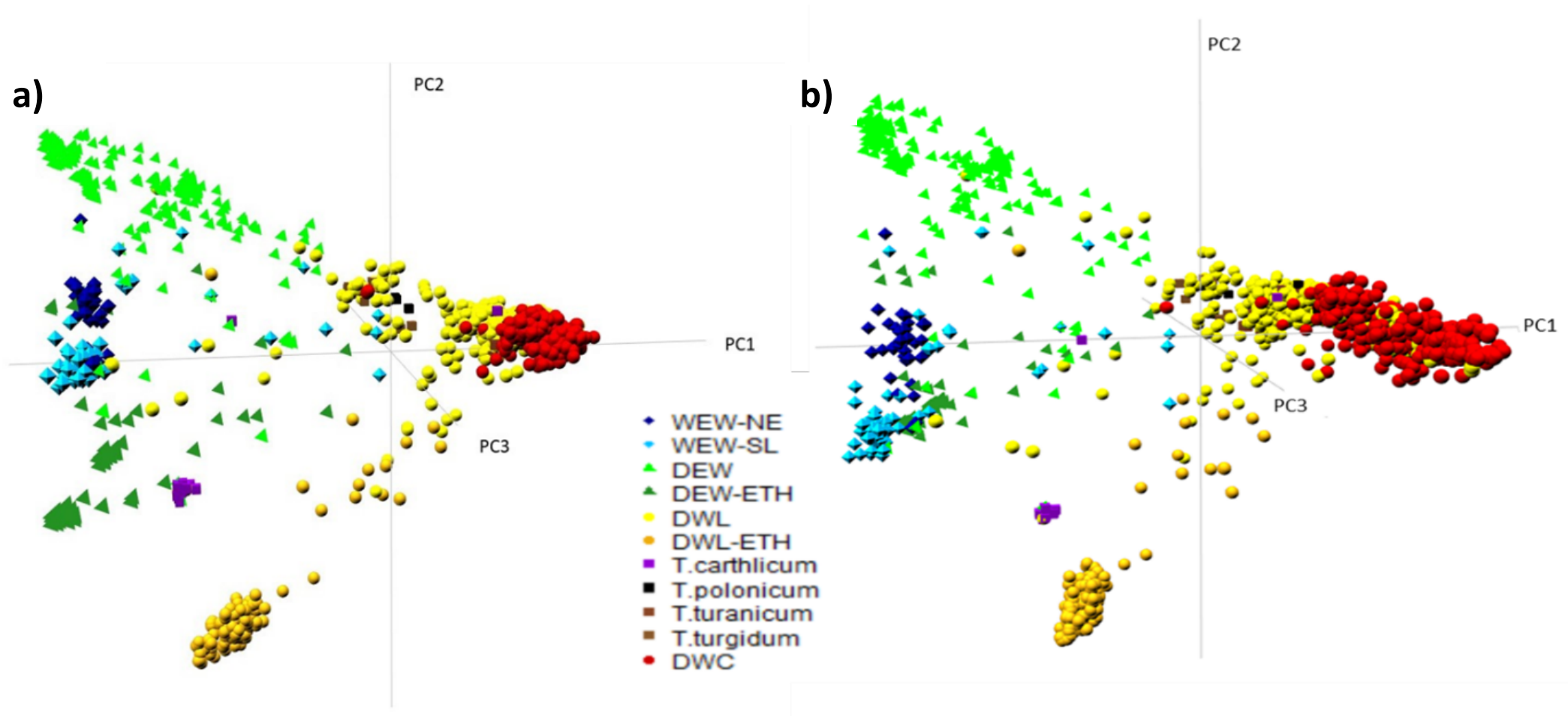

Supplement: Supplementary Figure 1 — Cumulative number of SNPs tagged by tSNPs at r2 ≥ 0.90 in each chromosome in wheat and barley. Curves are shown until the first singleton SNP is reached on each chromosome. [file Data_Sheet_1.zip › Supplementary Figure 2.PDF]

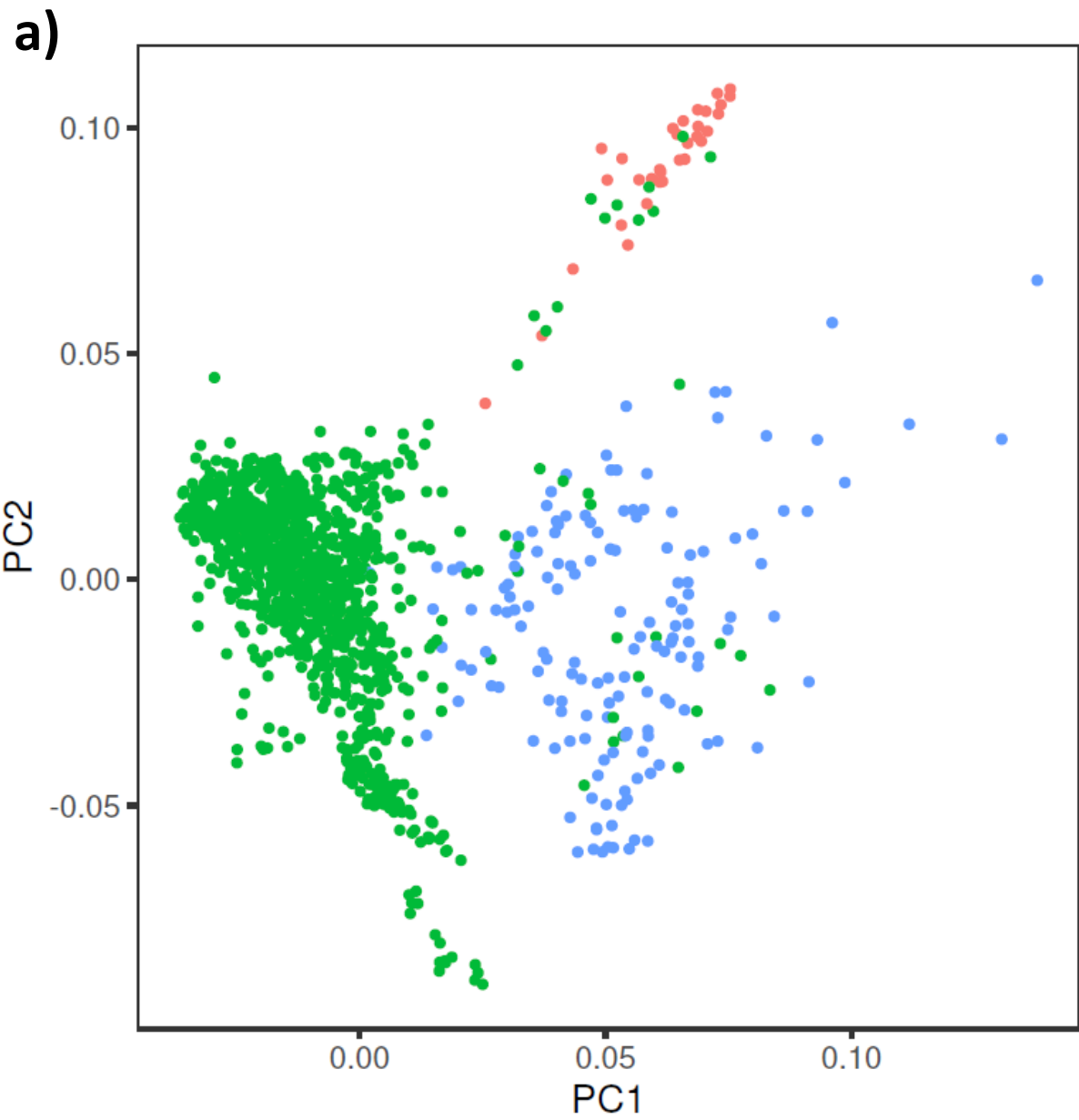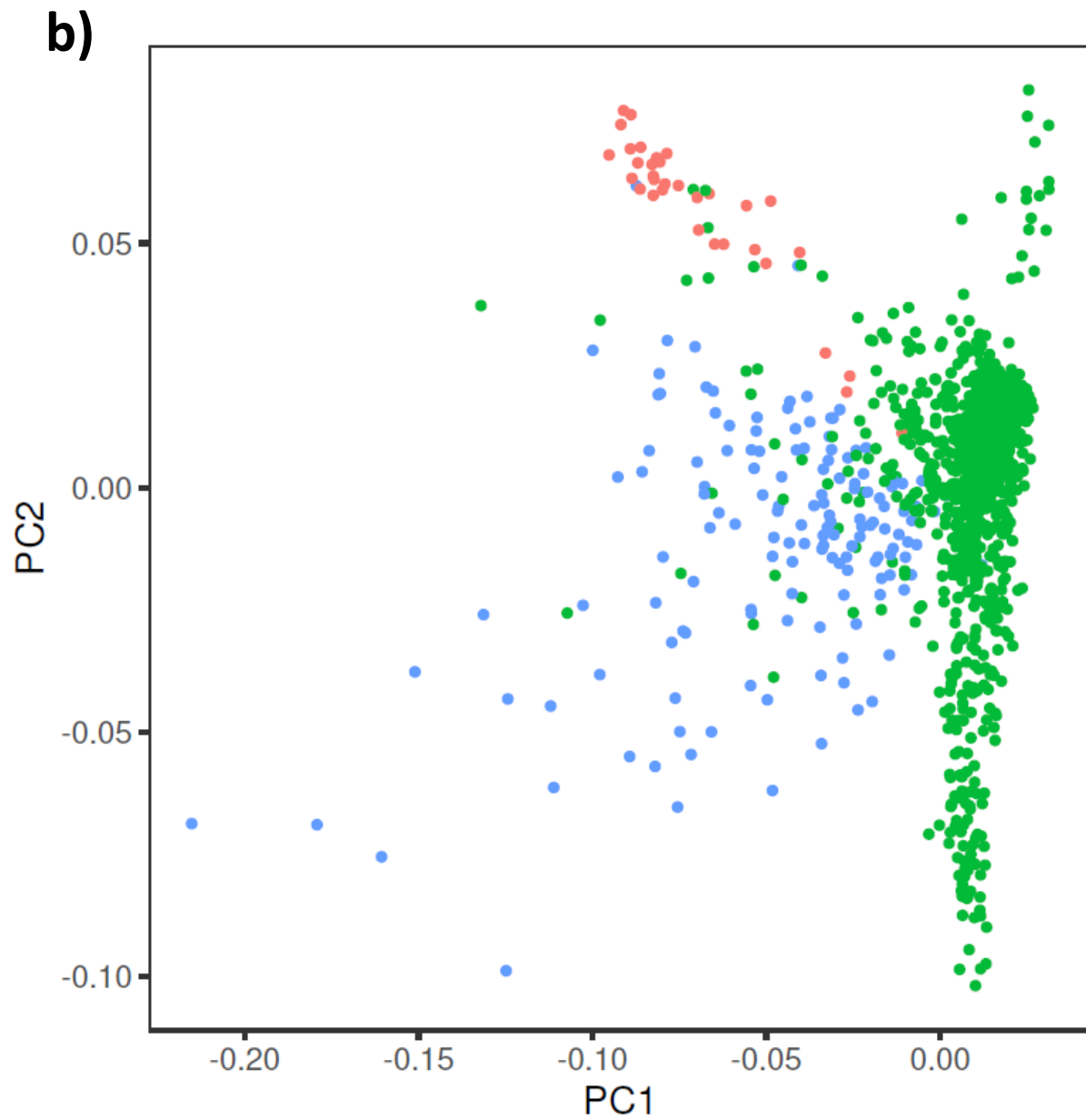

Supplement: Supplementary Figure 1 — Cumulative number of SNPs tagged by tSNPs at r2 ≥ 0.90 in each chromosome in wheat and barley. Curves are shown until the first singleton SNP is reached on each chromosome. [file Data_Sheet_1.zip › Supplementary Figure 3.PDF]

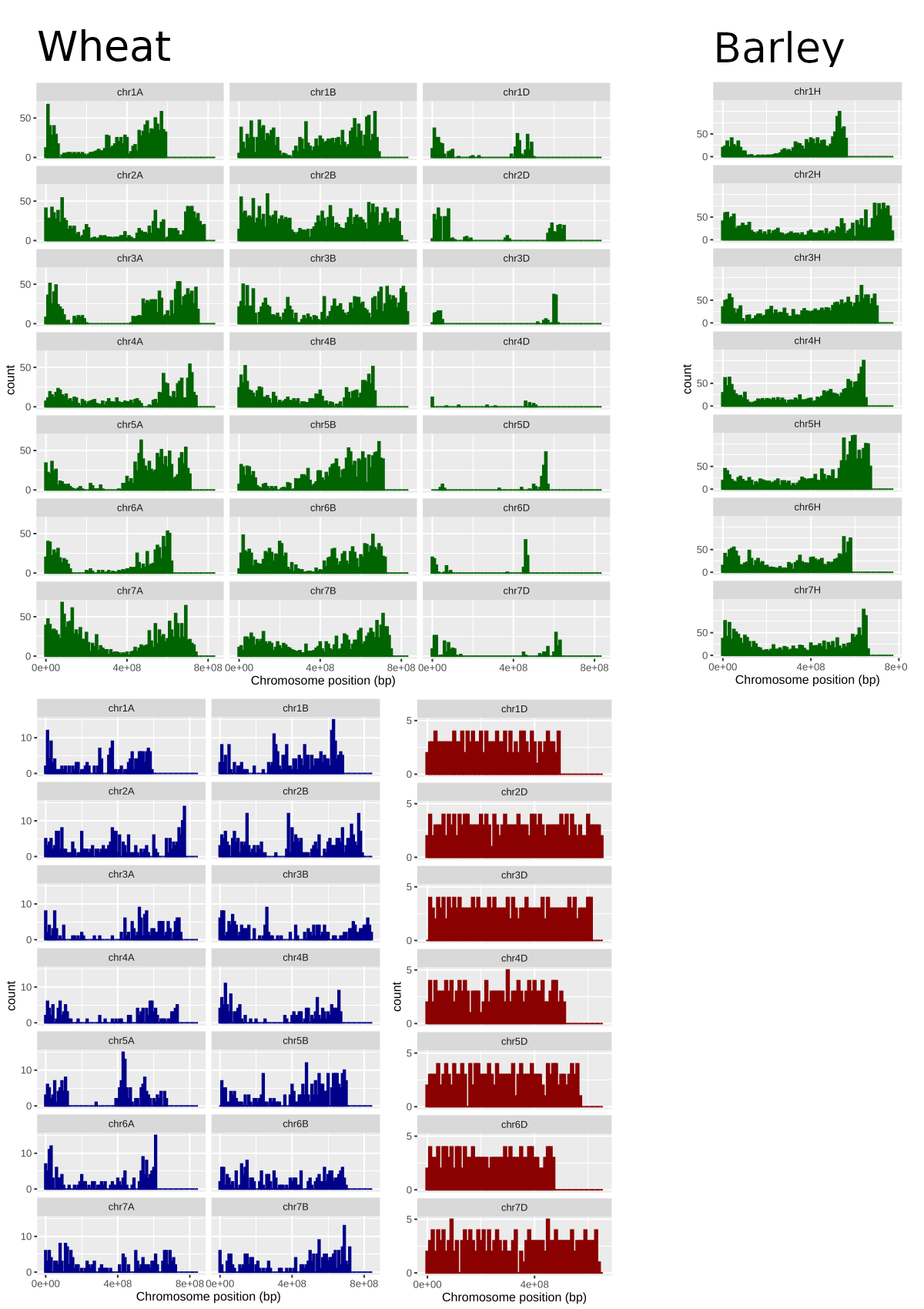

Supplement: Supplementary Figure 1 — Cumulative number of SNPs tagged by tSNPs at r2 ≥ 0.90 in each chromosome in wheat and barley. Curves are shown until the first singleton SNP is reached on each chromosome. [file Data_Sheet_1.zip › Supplementary Figure 4.TIF]

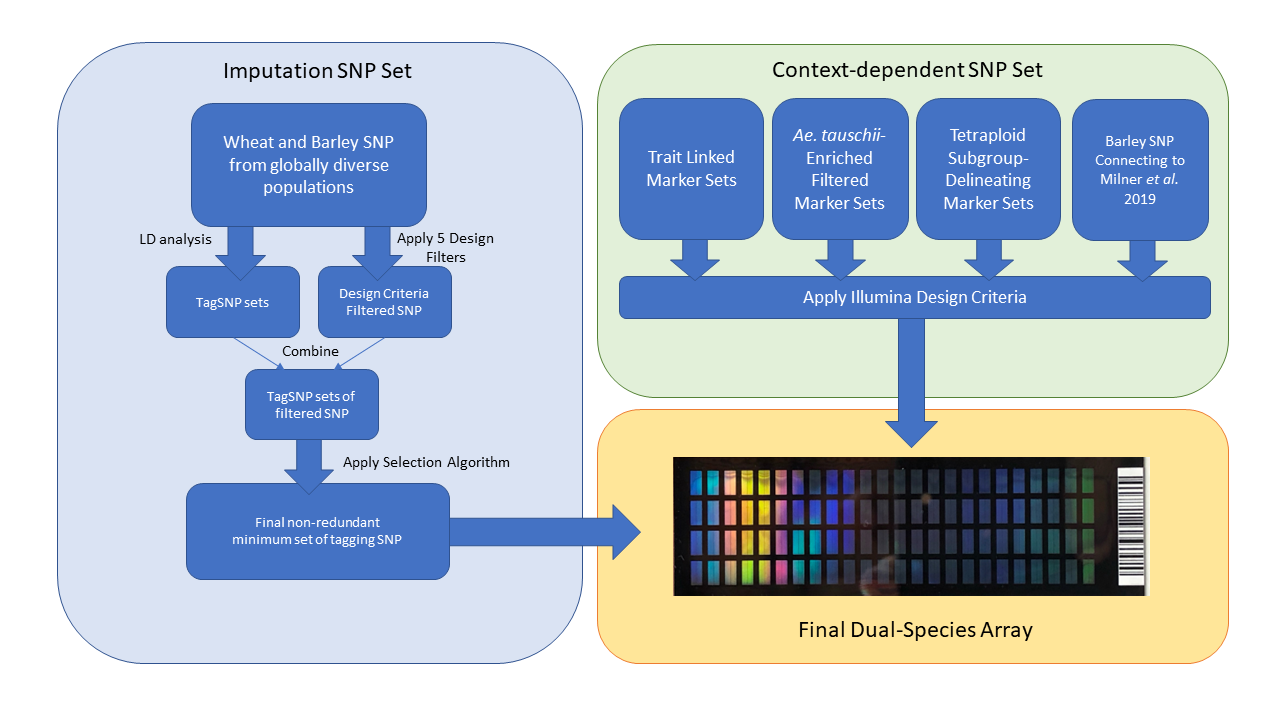

Supplement: Supplementary Figure 1 — Cumulative number of SNPs tagged by tSNPs at r2 ≥ 0.90 in each chromosome in wheat and barley. Curves are shown until the first singleton SNP is reached on each chromosome. [file Data_Sheet_1.zip › Supplementary Figure 5.TIF]
